# First isolation of *Mycobacterium brisbanense* from humans in the Americas

**DOI:** 10.1590/0074-02760250063

**Published:** 2025-10-24

**Authors:** Carlos Eduardo Dias Campos, Rachel dos Santos de Sena Vasconcelos, William Marco Vicente da Silva, Luciana Distásio de Carvalho, Melissa de Almeida Barbosa Eccard, Isabelle Pinheiro Nobre dos Santos, Jesus Pais Ramos

**Affiliations:** 1 Fundação Oswaldo Cruz-Fiocruz, Escola Nacional de Saúde Pública, Centro de Referência Professor Hélio Fraga, Laboratório de Referência Nacional de Tuberculose e Micobacterioses, Rio de Janeiro, RJ, Brasil

**Keywords:** Mycobacteria, M. brisbanense, drug susceptibility

## Abstract

**BACKGROUND:**

*Mycobacterium brisbanense* is a rare nontuberculous mycobacteria and was for the first time detected in the Americas in humans. However, this, like several other species of mycobacteria, may be underreported worldwide. Therefore, their study is increasingly important.

**OBJECTIVES:**

The aim of this article is to report and analyse the first seven human isolates of *M. brisbanense* in the Americas, derived from six Brazilian patients.

**METHODS:**

We sequenced the genes *hsp65*, *rpoB* and *16s rRNA*, of seven mycobacterial clinical isolates, constructed a phylogenetic tree, and determined their drug susceptibility profile.

**FINDINGS:**

The regions sequenced were highly similar between the *M. brisbanense* type strain and the Brazilian strains*.* Similarly, their susceptibility profiles were similar to that of *M. brisbanense* type strain, except for two antibiotics tested, cefoxitin and doxycycline.

**MAIN CONCLUSIONS:**

All studied strains were identified at the species level by a concatenated tree as *M. brisbanense*.

Currently, the genus *Mycobacterium* comprises 206 officially described species, which are either strictly pathogenic or opportunistic (https://www.bacterio.net/genus/mycobacterium). Excluding *Mycobacterium leprae* and those species classified in the *Mycobacterium tuberculosis* complex, nontuberculous mycobacteria (NTM) are classified into rapidly growing mycobacteria (RGM) and slowly growing mycobacteria (SGM) based on their growth rate on culture media. RGM forms visible colonies until seven days, whereas SGM requires more than seven days.^1^

*Mycobacterium fortuitum* was described in Brazil by Costa Cruz in 1938,^2^ isolated from a skin abscess in a patient after receiving subcutaneous injections of a vitamin preparation. This species was initially classified in two biovars, *M. fortuitum bv. fortuitum* and *M. fortuitum bv. peregrinum*.^3^ Wallace et al.^4^ studied a group of clinical strains of *M. fortuitum* and designated them as a *M. fortuitum* third biovariant complex. In 2004, Schinsky et al.^5^ named members of this complex as distinct species: *M. boenickei*, *M. houstonense*, *M. neworleansense*, and *M. brisbanense*.

*Mycobacterium brisbanense* can cause respiratory infections, soft tissue infections, bacteraemia, and disseminated disease.^6^ ^,^ ^7^ This species was isolated from an astral sinus in Australia.^5^ Five years later, again in Australia, a strain was isolated from herbicide-exposed soil.^8^ This organism was also detected in two patients with pulmonary infection: a 62-year-old and a 70-year-old, both from Malaysia.^7^ ^,^ ^9^ The first European case of lung infection in a 28-year-old patient with asthma was reported in Denmark.^6^ *M. brisbanense* may cause mycobacteriosis in animals, and strains were isolated from fish.^10^ *M. brisbanense* was also identified in Brazil from surface water samples collected from a zoological park.^11^

According to Schinsky et al.,^5^ based on the phylogenetic tree constructed with *16S rRNA* gene sequences, the species most closely related to *M. brisbanense* is *M. diernhoferi*. In contrast, Shahraki et al.^12^ later described *M. aquaticum* based on five Iranian isolates, which exhibited the highest *16S rRNA* gene sequence similarity to *M. brisbanense*.

Reports of rare or uncommon species have become increasingly frequent, so health professionals must be attentive to their diagnosis. To our knowledge, this is the first report of *M. brisbanense* human infection in a country on the American continent.

## SUBJECTS AND METHODS

This work includes seven strains isolated from six patients, sent to the National Reference Laboratory for Tuberculosis and Mycobacteriosis, CRPHF/ENSP/FIOCRUZ for identification at the species level between 2009 and 2023. Of these samples, four had pulmonary origin, two were from a surgical site, and one strain had unreported clinical material (Table I).

**TABLE I:** Strains characteristics

Strains	Patients	Year	Source	Geografic location
HF4555	1	2009	Sputum	Brazil
HF258284	2	2017	Surgical site	Brazil
HF212092	3	2019	Sputum	Brazil
HF212093	3	2019	Sputum	Brazil
HF252010	4	2020	Sputum	Brazil
HF421578803	5	2023	Surgical site	Brazil
HF150393164	6	2023	Not available	Brazil

*Sequencing* - The partial *16S rRNA*, *rpoB*, and *hsp65* genes were amplified as described respectively by Hall et al., Adékambi et al., and Telenti et al.^13^ ^,^ ^14^ ^,^ ^15^ Nucleotide sequences obtained were used in BLAST^16^ searches to identify similar sequences. For the analyses of the *16S rRNA*, *rpoB* and *hsp65* genes, we considered the cut-off values of ≥ 97, ≥ 98.3, and ≥ 99% respectively, for species identification.^14^ ^,^ ^17^ ^,^ ^18^

*Phylogenetic analysis* - The sequences were also used as input for building a concatenated phylogeny tree^19^ with the neighbour-joining method with MEGA (version 11).^20^ Node support in the neighbour-joining tree was assessed using 1 000 bootstrap replicates. Bootstrap values above 70% were considered indicative of well-supported branches.^21^ *M. tuberculosis* was used as an outgroup.

*Susceptibility tests* - Drugs susceptibility tests were performed according to Clinical and Laboratory Standards Institute (CLSI).^22^ ^,^ ^23^ The minimum inhibitory concentrations (MICs) were determined as lowest concentration of the drug that inhibited the visible growth of the isolates. The strains were grown in Lowenstein Jensen media for seven days, and colonies were diluted in water and adjusted to 0.5 McFarland standard. The microdilution assays were carried out with RAPMYCOI commercial plates (Thermo Scientific, Kansas, USA), which contain lyophilised antibiotics. They were prepared according to the manufacturer’s instructions for determination of MIC.

Plates were incubated for three to five days at 30ºC, and readings were taken using the Sensititre Vizion equipment. Drug susceptibility testing (DST) was performed for ten drugs according to CLSI,^23^ trimethoprim/Sulfamethoxazole, ciprofloxacin, moxifloxacin, cefoxitin, amikacin, doxycycline, tigecycline, clarithromycin, linezolid, imipenem, minocycline and tobramycin. The final reading for clarithromycin susceptible isolates was at 14th day to detect inducible resistance.

*Ethics* - This study was submitted to ethics committee of ENSP/FIOCRUZ (number 7.283.236). The ethics committee considered that this study should not be included as research involving human beings and, therefore, does not need to be evaluated by the CEP/Conep System.

## RESULTS

Table II shows accession numbers of type strains retrieved from GenBank and the accession numbers of sequences produced and deposited by this study.

**TABLE II:** Complete sequences used to construct the phylogenetic tree

Strains	GenBank accession numbers		
	*hsp65*	*rpoB*	16S rRNA
*Mycobacterium brisbanense* strain W6743	PQ241134.1	PP898190	NR_029037
*M. aquaticum* strain RW6	NR_158005	KY392539	NR_158005
*M. wolinskyi* ATCC700010	AY458064	AY262743	AY457083
*M. mageritense* CIP104973	AY458070	AY147169	NR_115232.1
*M. houstonense* ATCC49403	AY458077	AY147173.1	NR_042913
*M. conceptionense* CIP108544	AY859678	AY859695	AY859684
*M. senegalense* CIP104941	AY458067	JF706631	AY457081
*M. neworleansense* ATCC49404	AY458076	AY147172.1	AY457068
*M. boenickei* CIP107829	AY943195	AP022579.1	NR_029036
*M. diernhoferi* ATCC19340	AF547825	CP080332.1	NR_041903
*M. phocaicum* CIP108542	EU266579	EU254722	AY859682
*M. fortuitum* ATCC 6841	JF491295	JF346874	AJ536039
*M. smegmatis* ATCC 19420	AY458065.1	AY262735.1	AJ131761.1
*M. tuberculosis* H37RV	BX842573.1	BX842574.1	NR_102810
HF4555	PQ241130	PQ278250	PQ243596
HF258284	PQ241131	PQ278251	PQ243597
HF212092	PV360963	PV360964	PV350142
HF212093	PQ241132	PQ278252	PQ243598
HF252010	PQ241134	PQ278254	PQ243600
HF421578803	PQ241133	PQ278253	PQ243599
HF150393164	PQ241135	PQ278255	PQ243601

The basic local alignment search tool (BLAST) search showed that the *hsp65* 441-bp fragment of the seven strains is 100% identical to the *M. brisbanense* type strain or have only one mismatch with 99.75% of identity. The nearly complete *16S rRNA* gene of the strains exhibited identity varying from 99.25% to 99.75%. The *rpoB* gene showed the greatest variation, ranging from 98.33% to 99.72%.

Phylogenetic analyses were performed on an approximately 2,380 bp fragment comprising partial *16S rRNA*, *rpoB*, and *hsp65* sequences. The concatenated phylogenetic tree revealed that the strains tested clustered to the type strain of *M. brisbanense*, but are distinct from or all other sequences of type strains.

The seven strains were susceptible to amikacin, clarithromycin, linezolid and moxifloxacin. The DST profiles for ciprofloxacin, imipenem and trimethoprim/sulfamethoxazole varied, with susceptibility of 71%, 57% and 86%, respectively. Of the seven strains, six (86%) were resistant to doxycycline and tobramycin, while four (57%) were resistant to cefoxitin. The antimicrobial susceptibility profiles of the seven strains are presented in Table III.

**TABLE III:** Minimum inhibitory concentrations (MICs) (µg/mL) and susceptibility profiles of the seven strains

Antibiotic agent	HF4555	HF258284	HF212092	HF212093	HF252010	HF421578803	HF150393164
Amikacin	≤ 1(S)	2(S)	4(S)	≤ 1(S)	≤ 1(S)	1(S)	2(S)
Clarithromycin	1(S)	1(S)	0.5(S)	0.5(S)	0.5(S)	0.5(S)	0.5(S)
Imipenem	≤ 2(S)	8(I)	≤ 2(S)	4(S)	8(I)	4(S)	8(I)
Linezolid	2(S)	8(S)	2(S)	4(S)	4(S)	2(S)	4(S)
Cefoxitin	8(S)	128(R)	128(R)	128(R)	64(I)	64(I)	128(R)
Tobramycin	16(R)	8(R)	4(I)	8(R)	8(R)	8(R)	16(R)
Doxycycline	16(R)	16(R)	16(R)	16(R)	16(R)	0.12(S)	16(R)
Moxifloxacin	≤ 0.25(S)	0.5(S)	≤ 0.25(S)	≤ 0.25(S)	≤ 0.25(S)	≤ 0.25(S)	0.5(S)
Ciprofloxacin	0.25 (S)	1(S)	2(I)	2(I)	1(S)	1(S)	0.5(S)
Trimethoprim-sulfamethoxazole	1/19(S)	2/38(S)	2/38(S)	2/38(S)	8/152(R)	2/38(S)	1/19(S)
Tigecycline^*^	0.25	0.25	0.12	0.25	0.25	0.06	0.25
Minocycline^*^	8	8	8	8	8	1	8

^*^ There is currently no Clinical and Laboratory Standards Institute (CLSI) recommended breakpoint. I: intermediate; R: resistant; S: susceptible.

## DISCUSSION

We evaluated the antimycobacterial activity of 12 antibiotics against seven clinical isolates identified as *M. brisbanense*, in accordance with CLSI guidelines.^23^ Currently, data regarding the drug susceptibility profile of *M. brisbanense* remain limited, and this investigation constitutes the largest evaluation to date of clinical isolates of the species.

Our findings are consistent with those of Schinsky et al.,^5^ showing a high resistance rate to doxycycline and high susceptibility rates to amikacin, ciprofloxacin, and trimethoprim-sulfamethoxazole. Similarly, Poh et al.^7^ reported comparable results, with three isolates obtained from a single patient exhibiting susceptibility to amikacin, clarithromycin, and linezolid.

Consistent with the findings from our seven strains, Pang et al.^24^ reported similar susceptibility profiles for the reference strain *M. brisbanense* DSM 44680, which exhibited resistance to tobramycin and susceptibility to amikacin, clarithromycin, linezolid, and moxifloxacin. However, in contrast to our results, the reference strain demonstrated susceptibility to doxycycline.

Interestingly, the results obtained by Schinsky et al.^5^ and Pang et al.,^24^ mentioned above, differed in terms of susceptibility to doxycycline. Given that both studies used the same type strain, a similar susceptibility pattern would be expected. This discrepancy may be attributed to methodological differences in susceptibility testing. According to CLSI,^22^ microdilution assays, even when performed under standardized conditions, are subject to inherent variability, and identical breakpoints may not consistently yield reproducible results. Acceptable reproducibility is generally defined as a variation within one twofold dilution.

There are few data available in general on *M. brisbanense*, including those based on molecular biology, but our results were similar to those obtained by Mugetti et al.^10^ who showed 99.51% *hsp65* gene identity of four isolates with the type strain of *M. brisbanense*. The sequencing of the *hsp65*, *rpoB* and *16S rRNA* genes showed that all the isolates were within the cut-off established for the species level. Thus, all the identifications obtained by sequencing were considered valid.

As our seven strains and *M. brisbanense* type strain were included in a single clade with a high bootstrap value of 91, without including any other taxon, a monophyletic group was formed. Strains HF252010 and HF421578803 were the most distinct but still in the same clade of the type strain. The concatenated tree showed that *M. diernhoferi* type strain is not so close to *M. brisbanense*, as mentioned by Schinsky et al.^5^ This tree also shows the close relationship of *M. brisbanense* with *M. aquaticum* which corroborates the results obtained by Shahraki et al.^12^ (Figure). Phylogenetic and sequencing analysis confirmed that the seven isolates belong to the *M. brisbanense* species.

**Phylogenetic tree:**
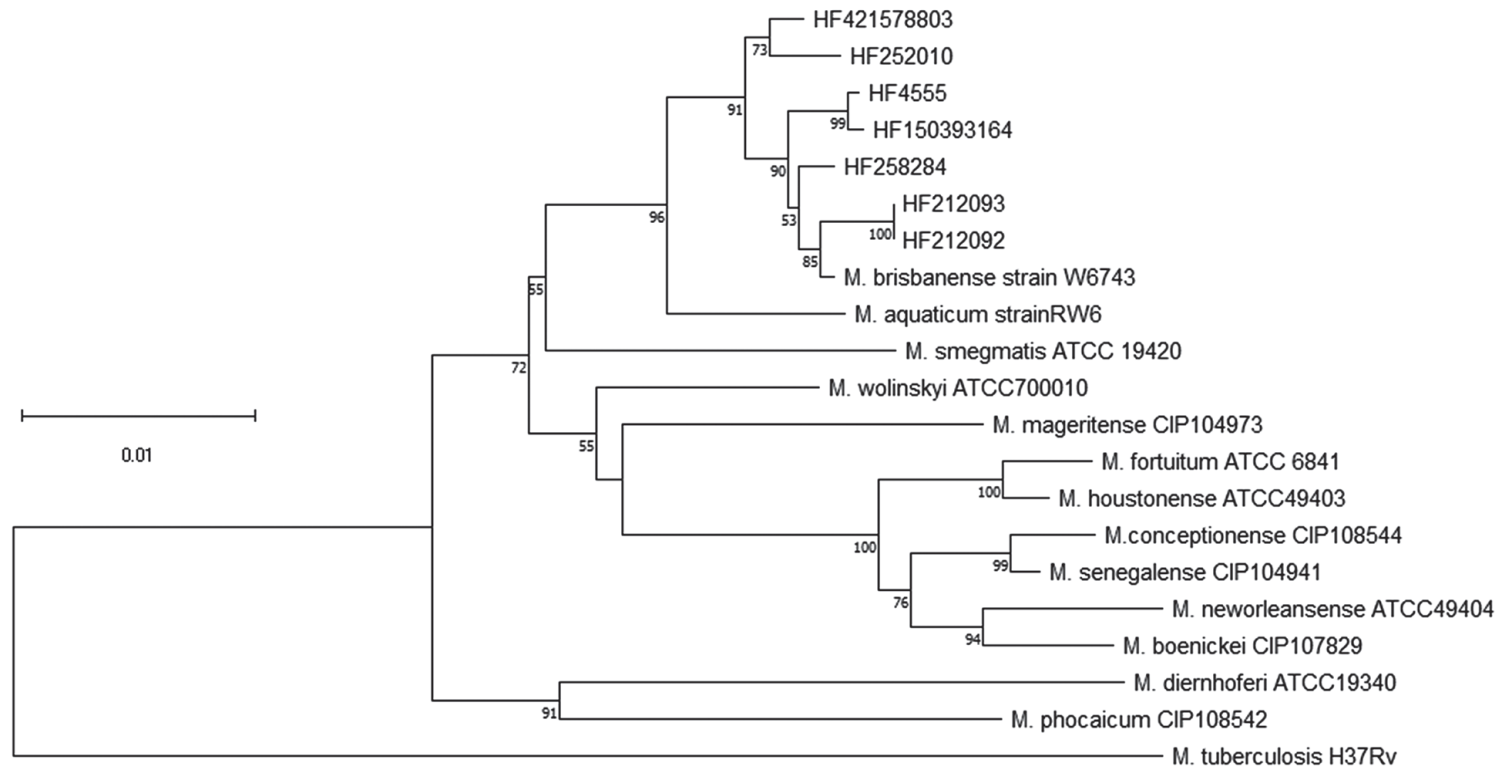
computed from the concatenation of *16S rRNA* gene, *rpoB*, and *hsp65* sequences by the neighbour-joining method and with Kimura’s two-parameter model as the substitution model. The significance of branches is indicated by bootstrap values calculated for 1000 replicates. *Mycobacterium tuberculosis* was used as an outgroup.

The number of diseases caused by NTM has been increasing worldwide. The most common NTM pathogens are *M. avium* complex, *M. kansasii*, *M. xenopi* and *M. abscessus* group, however other less frequent NTMs are of concern worldwide.^25^ Our results expand the possibility of new cases of *M. brisbanense* disease outside Europe/Asia/Oceania to countries of the American continent, such as Brazil.
